# Increasing Nucleosome Occupancy Is Correlated with an Increasing Mutation Rate so Long as DNA Repair Machinery Is Intact

**DOI:** 10.1371/journal.pone.0136574

**Published:** 2015-08-26

**Authors:** Puya G. Yazdi, Brian A. Pedersen, Jared F. Taylor, Omar S. Khattab, Yu-Han Chen, Yumay Chen, Steven E. Jacobsen, Ping H. Wang

**Affiliations:** 1 UC Irvine Diabetes Center, University of California at Irvine, Irvine, California, United States of America; 2 Sue and Bill Gross Stem Cell Research Center, University of California at Irvine, Irvine, California, United States of America; 3 Department of Medicine, University of California at Irvine, Irvine, California, United States of America; 4 Department of Biological Chemistry, University of California at Irvine, Irvine, California, United States of America; 5 Department of Physiology & Biophysics, University of California at Irvine, Irvine, California, United States of America; 6 Department of Molecular, Cell and Developmental Biology, University of California at Los Angeles, Los Angeles, California, United States of America; 7 Eli and Edythe Broad Center of Regenerative Medicine and Stem Cell Research, University of California at Los Angeles, Los Angeles, California, United States of America; 8 Howard Hughes Medical Institute, University of California at Los Angeles, Los Angeles, California, United States of America; Ludwig-Maximilians-Universität München, GERMANY

## Abstract

Deciphering the multitude of epigenomic and genomic factors that influence the mutation rate is an area of great interest in modern biology. Recently, chromatin has been shown to play a part in this process. To elucidate this relationship further, we integrated our own ultra-deep sequenced human nucleosomal DNA data set with a host of published human genomic and cancer genomic data sets. Our results revealed, that differences in nucleosome occupancy are associated with changes in base-specific mutation rates. Increasing nucleosome occupancy is associated with an increasing transition to transversion ratio and an increased germline mutation rate within the human genome. Additionally, cancer single nucleotide variants and microindels are enriched within nucleosomes and both the coding and non-coding cancer mutation rate increases with increasing nucleosome occupancy. There is an enrichment of cancer indels at the theoretical start (74 bp) and end (115 bp) of linker DNA between two nucleosomes. We then hypothesized that increasing nucleosome occupancy decreases access to DNA by DNA repair machinery and could account for the increasing mutation rate. Such a relationship should not exist in DNA repair knockouts, and we thus repeated our analysis in DNA repair machinery knockouts to test our hypothesis. Indeed, our results revealed no correlation between increasing nucleosome occupancy and increasing mutation rate in DNA repair knockouts. Our findings emphasize the linkage of the genome and epigenome through the nucleosome whose properties can affect genome evolution and genetic aberrations such as cancer.

## Introduction

With the advent of massively parallel DNA sequencing technologies it has become much easier to study and characterize somatic mutations and mutation rates across species[1]. Additionally, there are currently large projects underway attempting to catalog mutations responsible for the initiation and propagation of cancer[2–9]. These massive data sets represent some of the first and best sets for determining the various genomic and epigenomic factors that can affect mutation rates. Preliminary work has shown that various factors can affect regional mutation rates resulting in mutational heterogeneity. Of particular interest, recent work has shown that the mutation rate is strongly correlated with replication timing, transcriptional activity, and chromatin organization[10–12]. In eukaryotes, DNA is packaged into chromatin whose fundamental repeating unit is the nucleosome. Taken together, it is not surprising that previous work has demonstrated that nucleosome structure has played a role in human evolution[13]. Additionally, recent work in yeast has shown that nucleosome organization can affect base specific mutation rates[14]. In the context of the above, this study was carried out to further analyze the relationship between nucleosomes and mutation rates.

The nucleosome is comprised of two copies of each of the core histones (H2A, H2B, H3, and H4) wrapped around 147 base pairs (bp) of DNA, with the symmetrical center being called the dyad[15]. Besides being involved in packaging DNA, nucleosome positioning (the genomic location of nucleosomes), nucleosome occupancy (how enriched a genomic location is for nucleosomes), and epigenetic modifications (post-translational modifications of histones and DNA methylation) are thought to play a role in development, transcriptional regulation, cellular identity, evolution, and human disease[13, 16–24]. In order to determine its role in affecting mutation rates, we utilized paired-end sequenced Micrococcal Nuclease (MNase) digested DNA from H1 human embryonic stem cells (hESC), yielding ~180x depth of coverage of the human genome. A nucleosome occupancy score (NOS) map, at single bp resolution, was then calculated (Methods)[25]. Finally, this nucleosome data was analyzed against a diverse set of genomic features and data sets[1–9, 22, 26–31].

## Results

### Nucleosomes and human genetic variation, and mutations

**Fig 1:**
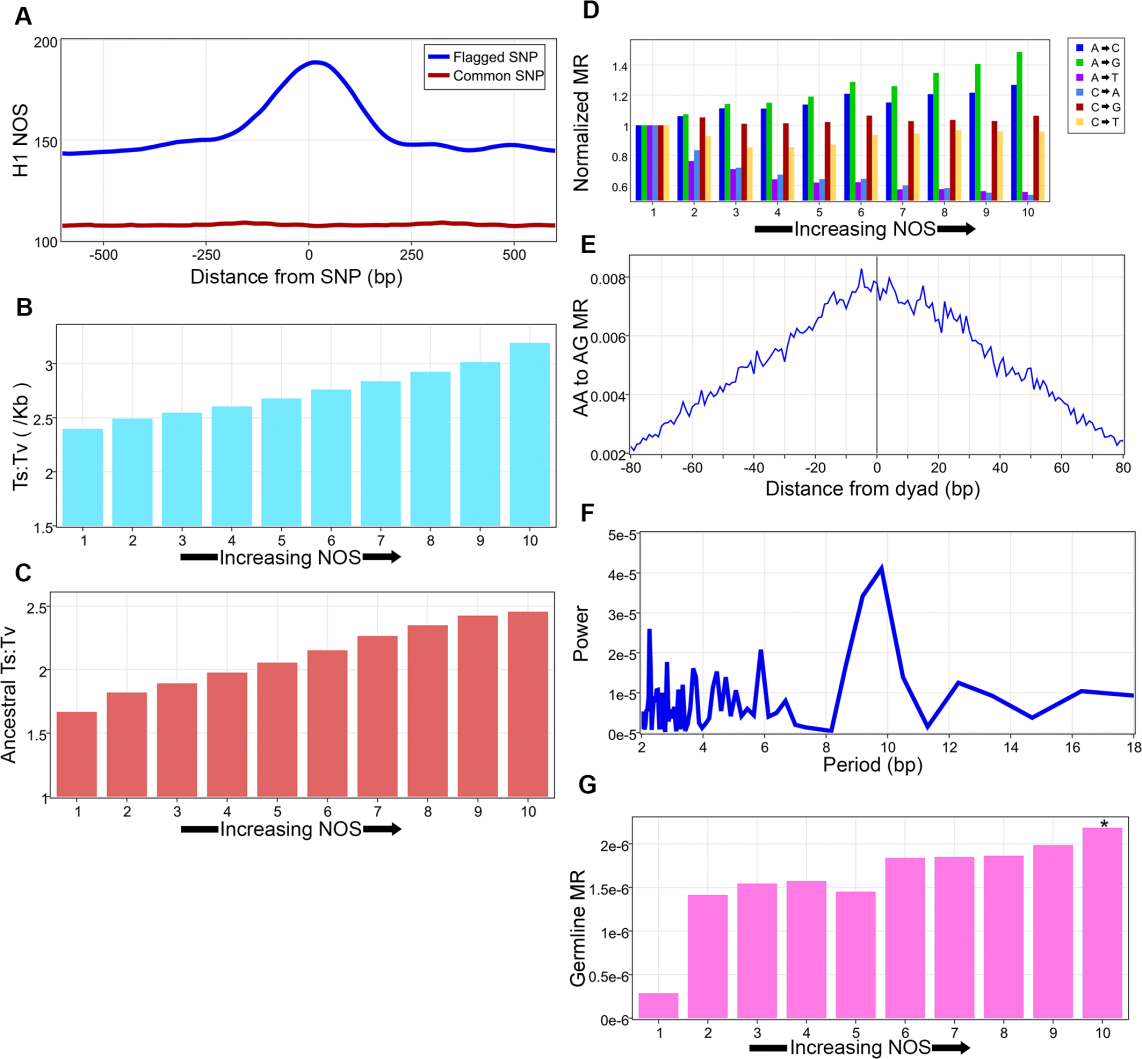
Nucleosomes and human genetic variation, and mutations. **A**, Nucleosome occupancy scores (NOS) around flagged SNPs and common SNPs. **B**, The average transition to transversion ratio in 1,000 bp bins as a function of NOS, calculated from 1,092 individuals. **C**, The ancestral transition to transversion ratio calculated for 10 groups corresponding to increasing nucleosome occupancy. **D**, Normalized base-specific mutation rates (MR) of 10 groups corresponding to increasing nucleosome occupancy. **E**, Ancestral AA→AG MR in relation to nearest dyad. **F**, Fast Fourier transform (FFT) of the AA→AG MR. **G**, Effect of increasing nucleosome occupancy on germline mutations, asterisk denotes statistical significance (p-value < 0.01 by Z-test with Bonferroni correction) between first and last group.

We sought to integrate our data with human genetic variation[29, 31]. Flagged single nucleotide polymorphisms (SNP) (SNPs deemed as potentially clinically significant with an allele frequency less than 1%) had an increased NOS in comparison to common SNPs (Fig 1A). By integrating genetic variation data from 1,092 individuals, we calculated average SNP densities, nucleotide diversity (π scores), and the transition to transversion (Ts:Tv) ratio in 1,000 bp bins for 10 equally sized groups of increasing nucleosome occupancy (Fig 1B, S1A and S1B Fig). Intrigued by the increase in the Ts:Tv ratio, the fact that nucleosomes in yeast can affect base-specific mutations, and the observation that on evolutionary time-scales SNPs are more likely to occur within nucleosomes while inversions and duplications are more likely to occur in nucleosome depleted regions (NDR), we sought to address the relationship between increasing nucleosome occupancy and the base-specific mutation rate (MR) in the human genome by strictly following previously used methodology[13, 14]. Our H1 single base pair resolution NOS map was used in all subsequent analyses. The ancestral genome was used to define mutations, with analyses kept to non-conserved, non-coding sites with high confidence ancestral allele information[1, 9]. Taking into account strand symmetry, we calculated the mutation rate for all 6 types of mutations (A→C, A→G, A→T, C→A, C→G, C→T) for 10 equally sized groups (bins) corresponding to increasing nucleosome occupancy. Nucleosomes suppress three types of mutations but are associated with increased mutations in the three others and an overall increased Ts:Tv ratio (Z-test with Bonferroni correction all p-values < 0.01, Fig 1C and 1D and S2A Fig). These findings are highly consistent with previous work in yeast[14]. Overall, the data demonstrates an increase in the mutation rate for nucleosome favoring DNA nucleotides as previous work by others has shown that the nucleosome core particle is enriched for Gs and Cs and relatively depleted of As and Ts[32]. This is consistent with recent work in yeast that observed selection against nucleosome favoring sequences in NDR and nucleosome disfavoring sequences in nucleosomal DNA[33]. The greatest overall increase was observed in the rate of change from A→G. Intrigued by the possibility that the structure of the nucleosome could be involved in this process, we analyzed the mutation rate at previously well described and evolutionary conserved DNA motifs within the nucleosome core particle. AA dinucleotides are an example of one such motif as they have been shown to be preferentially spaced approximately every 10bp at sites where the minor groove of DNA bends interiorly. As such, we calculated the AA→AG mutation rate and then plotted this rate for the highest NOS group against the closest dyad, revealing that it increases closer to the dyad (Fig 1E). Interestingly, the mutation rate displays a 10 bp periodic decrease away from the dyad, as calculated by fast Fourier transform (FFT) (Fig 1F). A Fourier transform is a mathematical method, with many different applications, that converts a signal in space into a combination of pure frequencies. As such, FFTs were performed for the AG dinucleotide to more precisely determine if a periodicity (1/frequency) existed, and if so what it is within the nucleosome core particle. This periodicity corresponds to the preferred 10 bp spacing of AA sites, as per theoretical rotational constraints[15]. We then became interested in the overall effect of nucleosome occupancy on mutation rates since this has not been previously done in humans. Calculating mutation rate as a function of nucleosome occupancy revealed a positive correlation of rate with NOS (Pearson’s correlation coefficient (PCC) = 0.817, S2B Fig). We repeated this analysis in yeast and found a similar result (data not shown). To further corroborate these findings we repeated our analysis, using the same methodology, on a germline mutation data set generated from an Icelandic population[30]. This same trend was found with germline mutations (Fig 1G).

### Nucleosome occupancy and cancer mutations

**Fig 2:**
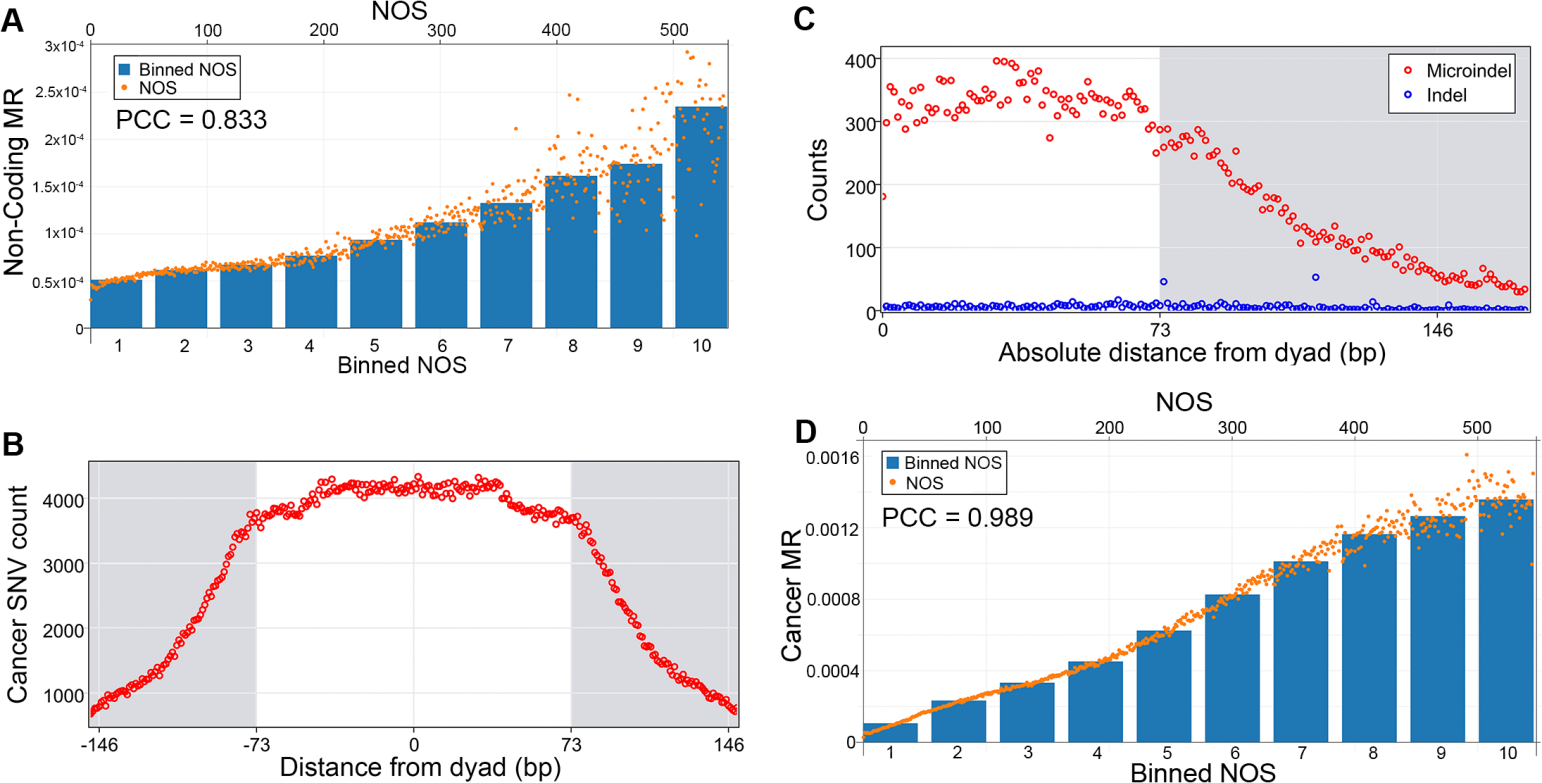
Nucleosome occupancy and cancer mutations. **A**, Cancer non-coding mutation rate (MR) in relation to nucleosome occupancy scores (NOS) with a Pearson’s correlation coefficient (PCC) of 0.833. Bottom x-axis corresponds to the bar graph depicting the NOS for 10 equally sized groups of increasing nucleosome occupancy. Top x-axis corresponds to the scatter plot depiction of the same data for each individual NOS. **B**, Raw counts of Cancer SNVs in relation to dyads. **C**, Cancer indel and microindel counts in relation to absolute distance to nearest dyad. Two small enrichments of indels are at 74 and 115 bp which correspond to the theoretical start and end locations of linker DNA between two nucleosomes. **D**, The total cancer (coding and non-coding) mutation rate as a function of NOS, Pearson’s correlation coefficient (PCC) of 0.989. Bottom x-axis corresponds to the bar graph depicting the NOS for 10 equally sized groups of increasing nucleosome occupancy. Top x-axis corresponds to the scatter plot depiction of the same data for each individual NOS.

We then hypothesized that nucleosome occupancy contributes to the heterogeneous nature of cancer mutations. As previously stated, currently there are major efforts underway to use sequencing technology to extensively catalog mutations involved in cancer[2–9]. Furthermore, one resulting conclusion from analyses of these studies is that the cancer mutation rate in the genome is heterogeneous[10]. The large size of these data sets allowed us to calculate these relationships at the level of a single base pair. Hence, in addition to repeating the binning analyses conducted previously, we directly analyzed mutation rates against NOS without binning. We find that the same mutation rate associations are observed within non-coding regions of cancers (PCC = 0.833, Fig 2A). Further characterization demonstrated cancer single nucleotide variants and microindels are enriched within nucleosomes, with a subset of indels being found at the theoretical start (74 bp) and end (115 bp) of linker DNA between two nucleosomes (Fig 2B and 2C). The total cancer mutation rate (non-coding and coding) is also highly correlated with increasing nucleosome occupancy (PCC = 0.989, Fig 2D). Finally, since huge genetic and epigenetic changes can occur in cancer which, in theory, could affect nucleosome occupancy, we sought to validate these findings by calling mutations in H1 cells directly. To this end, we conducted whole genome sequencing and called mutations in the same H1 cells we had used to generate our NOS map. We restricted our analysis to non-coding regions and found the same positive correlation between mutation rate and nucleosome occupancy (S3 Fig). Most interestingly, the PCC of this data set was highly similar to the somatic mutation dataset (0.854 for non-coding regions of H1 cells and 0.833 for the non-coding regions of cancers).

### Nucleosome occupancy and DNA repair

**Fig 3:**
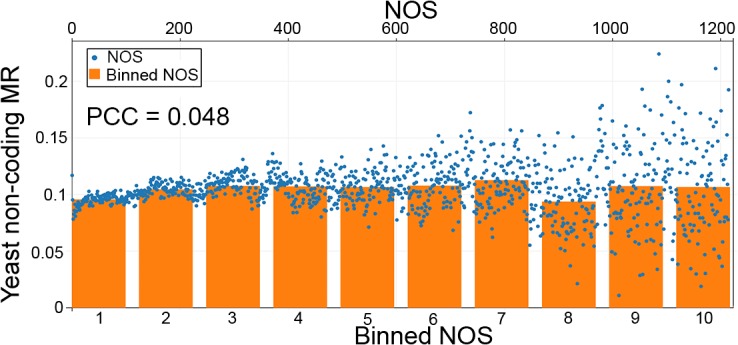
Effect of nucleosome occupancy on mutation rate in DNA repair deficient yeast. The non-coding mutation rate in yeast that lack DNA repair machinery in relation to nucleosome occupancy scores (NOS) with a Pearson’s correlation coefficient (PCC) of 0.048. Bottom x-axis corresponds to the bar graph depicting the NOS for 10 equally sized groups of increasing nucleosome occupancy. Top x-axis corresponds to the scatter plot depiction of the same data for each individual NOS.

These results are consistent with one of three possibilities: a confounding factor correlated with mutation rate which is also incidentally correlated with nucleosome occupancy; a biochemical mechanism mediated through nucleosomes which increases the number of mutations; and high nucleosome occupancy decreases access of the DNA mismatch repair machinery to DNA to fix replication errors and chemically modified nucleotides[34]. While it has been shown that nucleosomes do not entirely block access to the DNA repair machinery, this does not rule out that increased nucleosome occupancy can decrease efficiency of access, leading to an increased mutation rate as a result of less efficient repair[35]. Furthermore, our findings are highly consistent with this possibility since it would also explain our finding that the overall mutation trend is toward more nucleosome favoring bases. In order to test our hypothesis, we used a large data set of yeast DNA repair machinery knockouts consisting of 16 different mutant yeast strains to calculate mutation rates and analyzed it against yeast NOS[36–38]. This data demonstrated no correlation between mutation rate and nucleosome occupancy (Fig 3). Overall, these results are consistent with a model in which increasing nucleosome occupancy decreases access of DNA repair machinery to DNA, resulting in an increased mutation rate.

## Discussion

We sought to understand the role nucleosomes play in affecting mutation rates, especially as it relates to human cancer and genome evolution. Previous work looking at potential epigenomic or chromatin effects has been done on kilo- or megabase scales. By utilizing our ~180x depth of coverage nucleosome map, our analyses allowed us to analyze this relationship at single base pair resolution. We first integrated our data with genetic variation data. Most interestingly, we found an increasing transition to transversion ratio with increasing nucleosome occupancy. This was revealed by analyzing 1000 Genomes data and the ancestral genome in conjunction with our NOS map. This implies that these associations are related to DNA / histone interactions and not just a result of sequencing biases or biases in the 1000 Genomes data set. We kept our analyses to non-coding and non-conserved sites by excluding all areas under mammalian conservation[1]. By calculating base-specific mutation rates from the ancestral genome, we found that increasing nucleosome occupancy is associated with rate changes that are consistent with changes that would select for nucleotides which are favored within nucleosomes.

Under normal physiological conditions, DNA can locally denature to become single stranded. This concept is termed “DNA breathing”[39, 40]. This phenomenon is important as “open” or “breathing” regions of DNA are more chemically reactive in comparison to those that are in a double helix. Importantly, the likelihood of a region of DNA to be breathing is inversely proportional to the nucleosome occupancy of that region (the higher the nucleosome occupancy, the lower the likelihood for a region to be breathing). As the different DNA bases have unique chemical reactivities, the nucleotide frequencies within the nucleosome core particle will also influence the mutation rate as a function of nucleosome occupancy. Conversely, there is a selective pressure against bases that are less favored within nucleosomes. The AA→AG mutation rate also corroborates this finding by demonstrating a periodicity within the nucleosome and decreasing at sites corresponding to preferred AA sites within the nucleosome core particle. Previous work demonstrates that nucleosomal DNA has an enriched G/C content[32, 41, 42]. In the context of these attributes, one would expect the absolute mutation rate of the different mutation types to reflect this. This can appreciated with our data.

We have recently demonstrated that DNA methylation is associated with increasing nucleosome occupancy in the human genome, and in the context that methylcytosines are more likely to undergo spontaneous deamination in comparison to cytosines, we believe that the latter increase in the C to T rate at higher nucleosome occupancies is due to methylated cytosines[41, 43]. The two types of mutations with the highest absolute baseline mutation rate (rate within bin “1”) are C→T and A→G. These two transition mutations are the most commonly observed mutations in genomes and can be caused by oxidative deamination of Cs and oxidative deamination and tautomerization of As[44]. Given the mechanism of these changes, one would expect a decreasing mutation rate as the NOS increases as this would permit for less DNA breathing and thus less reactivity. The opposite of this was observed for A→G mutations and thus led to our hypothesized mechanism.

Suppression of the mutation rate was observed for C→T mutations. However the decrease is perhaps not as much as one would predict given the previously observed decrease in *S*. *cerevisiae*[14]. This can perhaps be explained in part by the increased spontaneous deamination of 5-methylcytosine in comparison to unmethylated cytosines, and that *S*. *cerevisiae* has relatively few 5-methylcytosines[43, 45]. In addition, increasing 5-methylcytosine content within the nucleosome core particle was correlated with increasing nucleosome occupancy[41]. The decreased mutation rate for C→T mutations in humans as a function of nucleosome occupancy is thus perhaps attenuated by the increased content of 5-methylcytosines in regions with a high nucleosome occupancy.

C→A mutations were the third most common type of mutation at the lowest nucleosome occupancy level. Interestingly, this type of mutation had the greatest fold reduction with increasing nucleosome occupancy. This type of transversion mutation can arise when guanine residues undergo oxidation to become 8-oxoguanine that can then form a Hoogsteen base pairing with adenine[46]. This mismatching can result in G→T substitutions by DNA repair machinery and thus C→A mutations[47]. 2-hydroxyadenine arises when adenine residues undergo oxidation[48]. Previously studies have demonstrated that DNA polymerases can incorporate dAMP opposite 2-hydroxyadenine and thus introduce A→T mutations[49]. With increasing nucleosome occupancy, one would expect less DNA breathing and thus a decreased susceptibility of guanines and adenines to these oxidation reactions and thus C→A and A→T mutations, respectively.

Previous work has indicated a selective pressure for an increase in nucleosome favoring DNA sequences[50, 51]. In particular, G/C rich regions are more likely to be associated with increased nucleosome occupancy. Additionally, CC/CG/GC/GG dinucleotides are favored in locations where the minor groove faces away from the histone surface and AA/AT/TA/TT dinucleotides are favored where the minor groove is directed towards the surface of the histones. These selective forces may contribute to the increasing A→C mutation rate as a function of increasing nucleosome occupancy.

The absolute mutation rate of C→G varies the least for all of the different types of mutations. Of note, the lowest mutation rate for this type of mutation was observed for regions with the lowest nucleosome occupancy and was then increased but relatively invariably and marginally. Since nucleosomes favor both Gs and Cs within their core, the C→G mutation rate should be less affected by changes in nucleosome occupancy and the slight increased mutation rate with increasing nucleosome occupancy is probably largely a function of an increased G/C content within the nucleosome core particle

Overall, these findings strongly imply that the DNA sequence preferences within the core particle have had an impact on the evolution of the human genome. This is demonstrated by DNA sequences drifting over time to nucleotide compositions that are more favored by nucleosomes, especially in areas characterized by high nucleosome occupancy sans natural selection pressure. These findings are consistent with initial evolutionary analyses and especially with work done in yeast[50].

We then became interested in deducing the overall effect of nucleosome occupancy on mutation rate. When all base specific rates were analyzed together, we found that increasing nucleosome occupancy was associated with an increasing mutation rate. We corroborated this conclusion by performing the same analysis using germline mutation data from an Icelandic population.

To test this correlation on the somatic cell mutation rate, we turned our attention to the cancer mutation rate as the abundance of sequencing data sets can be used to test these associations. We sought to address mutational heterogeneity as a function of nucleosome occupancy as this heterogeneity represents a substantial problem in cancer genomics. In cancer, the coding and non-coding mutation rate increased with increasing nucleosome occupancy. Interestingly, the PCC of the cancer non-coding mutation rate was highly similar to the PCC of the ancestral mutation rate (0.833 and 0.817, respectively), implying that these associations are related to DNA / histone interactions and not artifacts of the mutation data sets used. Additionally, we repeated this analysis by calling mutations in H1 cells directly and found the same positive correlation between mutation rate and nucleosome occupancy. This falls in line with our current unpublished work and previous work that has demonstrated that on a global level nucleosome occupancies are correlated between different cell types[52].

While it is interesting to note that the data appears to show that the germline mutation rate is lower in the first binned group than the mutation rate observed for the lowest somatic mutation groups, it must be stated that the germline mutation rate analysis was generated by binning NOS into 10 equal size bins and is not a direct comparison of mutation rate to NOS. This was done because the germline mutations were very few in number, 4,934 to be exact[30]. Hence, there were not enough data points to accurately quantify the mutation rate for every corresponding NOS score. Additionally, due to the limited nature of the germline data set, making direct comparisons to the somatic data set is difficult due to the fact that the cancer mutation data is comprised of hundreds of data sets. For us, the bigger point, which the data does show, is that the same overall trend is observed in the germline data set. In the future, it would be of interest to find out if there is a difference between germline and somatic mutation rates as it relates to low nucleosome occupancy and what could be potentially driving that variability. Overall, we can surmise that variations in nucleosome occupancy can account for a large proportion of the mutation rate variation in the genome.

While microindels behaved like cancer single nucleotide mutations in relation to nucleosome occupancy, indels were increased at 74 and 115 bp from the dyad, which correspond to the theoretical entry sites of DNA in the linker region between two nucleosomes. These findings suggest that nucleosome architecture can have a substantial impact on cancer mutations by increasing mutation rate within the core particle and influencing the sites of insertions, deletions, and duplications. This is in line with recent data from the Roadmap Epigenomics Project, which demonstrated cell-type specific cancer mutations are influenced by cell-type specific chromatin architecture[53]. Future studies integrating nucleosome occupancy data into mathematical models of cancer genomics may better determine which aberrations are cancer driver mutations.

Finally, we sought to explore a potential mechanism that could explain these findings. Three of the potential mechanisms that could explain our findings are: nucleosome occupancy is associated with another parameter responsible for mutations; nucleosomes biochemically increase mutations; and/or increasing nucleosome occupancy decreases access of DNA repair machinery to DNA, thereby increasing the rate of mutation by decreasing the efficiency of repair. The third possibility seemed most likely based on the totality of our data. The most convincing evidence of this is our findings that, over time, the human genome seems to drift towards nucleosome favoring sequences and the near linear relationship between nucleosome occupancy and mutation rate. In order to test for this possibility, we repeated our analyses using 16 different large data sets from yeast DNA repair knockout strains. In order to eliminate as much bias as possible, we conducted the analysis in the non-coding regions only. Yeast coding mutations were excluded from the final analysis for the following two reasons. First, coding mutations can alter phenotype and therefore be associated with a corresponding change in fitness. As such, variations in selective pressure can alter or bias any analysis of mutation rates. We calculated the non-coding mutation rate for all data sets, because, in theory, these mutations are not under selective pressure that can alter or bias their calculations. Second, it is well known that coding regions have higher nucleosome occupancy than non-coding regions[54, 55]. This could significantly bias any analysis on nucleosome occupancy and mutation rates. The main point of our yeast analysis was to demonstrate the loss of this correlation with DNA repair knockouts. In fact, in these strains there was no correlation between nucleosome occupancy and mutation rate. Future biochemical studies are needed to shed light on the exact nature of the interaction between nucleosomes and DNA repair proteins.

In summary, our analyses have revealed that mutation rates are affected by nucleosome occupancy so long as DNA repair machinery remains intact. This association has significantly impacted genome evolution and cancer mutagenesis. Finally, this relationship can partially explain the heterogeneous nature of cancer mutations. Going forward, it will be interesting to integrate this relationship into mathematical models of cancer, with the aim of developing better tools for determining which mutations are driving cancer pathophysiology.

## Materials and Methods

### Cell culture

The UC Irvine Human Stem Cell Research Oversight Committee (UCI hSCRO) approved the use of human embryonic stem cells in this study. The H1 human embryonic stem cell line was purchased from WiCell Research Institute, Inc. This one of the first ever human embryonic stem cell lines derived and are approved by the NIH Human Embryonic Stem Cell Registry (http://grants.nih.gov/stem_cells/registry/current.htm) [56]. The NIH Registration Number for H1human embryonic stem cells is 0043. Feeder free cultures of H1 human embryonic stem cells were grown and passaged in mTeSR 1 (STEMCELL Technologies Inc) as previously described and in accordance with ENCODE protocols[26]. In total, approximately 100 million H1 cells corresponding to passages 33–35 were used in experiments.

### Generation of mono-nucleosomal DNA sequenced reads

H1 cells were subjected to MNase digestion by use of the EZ Nucleosomal DNA Kit (Zymo Research) in accordance with the manufacturer’s protocol. The ideal digestion should yield approximately 80% mono-nucleosomal DNA[17–20]. In order to extract both easily digested nucleosomes and less digestible ones, we titrated the time of digestion in multiple replicates to yield 70% to 90% mono-nucleosomal DNA, with the average being 80% from all replicates combined. We then prepared paired-end libraries from this total mono-nucleosomal DNA with use of the Illumina Paired-End DNA Sample Prep Kit according to the manufacturer’s instructions with the following exception. In order to reduce potential PCR amplification bias, we performed two separate PCR reaction steps and combined the product of the two reactions[57, 58]. The libraries were then sequenced using PE54 chemistry on the Illumina HiSeq2000 in replicate on two flow cells (R51 and R54). Two biological replicates for H1 were performed, each consisting of six technical replicates.

### Alignment and processing of nucleosome maps

Paired-end nucleosomal sequencing data for R54 was aligned to the hg19 reference genome using Bowtie 2 on default settings[59]. Data from R51 was processed similarly with the exception that 25 bases from the 3' end of read 2 were removed as these final cycles produced low Q-scores which caused excess reads to not align properly. All aligned data was processed using SAMtools to yield merged BAM files[60].

### Nucleosome occupancy score map generation and calling nucleosomes

BAM files were run through the DANPOS algorithm in which reads were clonally cut to remove potential PCR amplification bias, smoothed, and adjusted for nucleosome size to enhance signal to noise ratio, resulting in a nucleosome occupancy score (NOS) for each base in the human genome[25]. DANPOS settings were as follows:-d 150,-a 1,-k 1,-e 1,--paired 1.-d 150 denoted setting the minimal distance between nucleosome dyads to 150 bp. The distance between dyads was set to 150 bp as the average fragment size from our H1 paired-end sequencing dataset was 151 bp (corresponding to 75 bp on either side of a dyad). -a 1 set the resolution of the NOS maps at a single bp and thus obviated any further downstream signal smoothing. The setting -e 1 allows for an edge-finding step to be taken, which estimates the edges of the predicted nucleosomes. -k 1 led to all data from intermediate steps being saved.--paired 1 indicated that the input BAM files were from paired-end sequencing data. We also generated NOS and called nucleosomes for the H1 dataset corrected for MNase digestion bias with use of a genomic control and found no significant differences in sequence preference analyses (data not shown)[32, 36, 41, 61]. For all subsequent analyses we used our original NOS map.

### General software used for analysis

Operations on genomic intervals were performed using BEDTools[62]. Fast Fourier transforms were done using MATLAB. Statistics were done in R. Additionally, we made use of in-house Python 2.7, C++, and shell scripts that are available upon request.

### Genetic variation

Flagged and common SNP data were downloaded from the UCSC genome browser table[31, 63]. 1000 Genomes data was downloaded and processed using the VCFtools software package to calculate SNP densities, pi scores, and the transition to transversion ratio in 1,000 bp bins[64]. For the same bins, we also calculated average H1 NOS. We then partitioned our data into ten equal sized groups corresponding to increasing average NOS and all parameters were averaged within these groups.

### Base-specific and total ancestral mutation rate

To ease downstream analysis we took our single bp resolution H1 NOS map and rounded all NOS values to the nearest whole integer and replaced all values above 545 as 545. This new whole integer NOS map was used for all subsequent analyses. We then downloaded coordinates of conserved elements in the human genome and aligned them to hg19 using liftOver[1, 63]. We combined these coordinates with gene coordinates from RefSeq and ENCODE blacklist regions[26, 65]. We used these combined coordinates to remove all conserved, blacklist, and coding sites from our NOS map. Using the ancestral genome from Ensembl, we calculated the ancestral allele and the current allele for all remaining sites taking into account strand symmetry[9]. Additionally, we removed all sites without known ancestral or current allele information and kept the analysis to sites in which the ancestral allele had a high confidence call according to Ensembl. In total, after this filtering, we ended up with greater than 2 billion bases. For each ancestral base, A or C, the data was broken up into 10 roughly equal sized groups corresponding to increasing NOS. Base-specific MR were calculated for each group as the number of base-specific mutations divided by the total number of bases. Finally, we calculated a total NOS specific MR by dividing the number of mutations by the total number of bases found for each whole integer NOS of 0–545.

### Germline and cancer mutations

Germline mutations were downloaded as processed mutation calls with gene coordinates from supplementary information and converted to hg19 by use of liftOver[30, 63]. We broke up the human genome into 10 roughly equal sized groups of increasing NOS. The germline mutation rate was calculated for each group by taking the total number of germline mutations in that group and dividing it by the total number of bases. For cancer mutations, we downloaded processed mutation data from six Cancer Genome Atlas Research Network studies and The Catalog of Somatic Mutations in Cancer (COSMIC) and combined all the mutations with two caveats[2–8]. First, we kept the analysis to one single nucleotide variant (SNV) per genomic coordinate for each cancer type, regardless of the frequency of a mutation within a cancer. SNVs in different cancers were analyzed as multiple mutations per genomic coordinate with the amount of mutations equaling the number of different cancers they were found in. For indels and microindels this same approach was used but for an indel to be excluded both the start and end coordinates must have been the same. Indel and microindel start and stop coordinates were used as the genomic coordinates of the mutation and all bases that fell within these coordinates were ignored for downstream analysis. All insertion or deletion mutations 50 bp or greater were called indels and all those greater than 1 bp but less than 50 were called microindels. As before, to ease downstream analysis we took our single bp resolution H1 NOS map and rounded all NOS values to the nearest whole integer and replaced all values above 545 as 545. From this map we assigned a NOS to each cancer mutation. We initially extracted just the non-coding mutations and calculated NOS specific cancer non-coding MR as the total number of non-coding mutations divided by the total number of non-coding bases found for each whole integer NOS of 0–545. The total cancer mutation rate was calculated as the total number of cancer mutations (coding and non-coding) divided by the total number of bases found for each whole integer NOS of 0–545.

### H1 genome sequencing and analysis

We prepared paired-end libraries from H1 DNA with the use of the Illumina Paired-End DNA Sample Prep Kit according to the manufacturer’s instructions with the following exception. In order to reduce potential PCR amplification bias, we performed two separate PCR reaction steps and combined the product of the two reactions[57, 58]. The libraries were then sequenced using PE150 chemistry on the Illumina HiSeq2500 in replicate on two flow cells (R170 and R171). Two biological replicates for H1 were performed, each consisting of two technical replicates. In accordance with GATK Best Practices, this data was processed using base quality score recalibration, indel realignment, duplicate removal, SNP and INDEL discovery, standard hard filtering parameters, and variant quality score recalibration[66–69]. Called mutations were processed as above.

### Yeast DNA repair knockout strains and mutations

We downloaded yeast genomic sequencing data from 16 mismatch repair knockouts, one control, and MNase-Seq data from the NCBI[19, 37, 38, 70]. The yeast MNase-Seq data was processed through the same pipeline stated above. Using VarScan 2, mutations for all 16 knockouts were called against the control strain[71]. We kept our subsequent analysis to non-coding mutations and calculated mutation rates against yeast NOS, exactly as done for the human data as stated above.

## Supporting Information

S1 Fig Mutation density and nucleotide diversity as a function of NOS. **A**, The average SNP density was calculated as the number of SNPs per 1,000 bp then averaged for each equal sized group corresponding to increasing nucleosome occupancy. Genetic variation data was generated from The 1000 Genomes Project. **B**, Groups 1–10 correspond to groups with increasing nucleosome occupancy scores (NOS). The π score is a measure of nucleotide diversity and was calculated in 1,000 bp bins.
(TIF)

S2 Fig Base-specific and overall mutation rate as a function of increasing NOS. **A**, Ancestral base-specific mutation rates (MR) calculated for ten equally sized groups corresponding to increasing nucleosome occupancy scores (NOS) with color coded legend for the type of mutation at top, with asterisks denoting statistical significance (p-value < 0.01) between the first and last group. **B**, Ancestral MR in relation to nucleosome occupancy with a Pearson’s correlation coefficient (PCC) of 0.817.
(TIF)

S3 Fig H1 mutation rate (MR) as a function of nucleosome occupancy. Bottom x-axis corresponds to the bar graph depicting the NOS for 10 equally sized groups of increasing nucleosome occupancy. Top x-axis corresponds to the scatter plot depiction of the same data for each individual NOS. Pearson’s correlation coefficient (PCC) of 0.833.
(TIF)

## References

[1] Lindblad-Toh K, Garber M, Zuk O, Lin MF, Parker BJ, Washietl S, et al A high-resolution map of human evolutionary constraint using 29 mammals. Nature. 2011;478(7370):476–82. 10.1038/nature10530 21993624 PMC3207357

[2] Forbes SA, Tang G, Bindal N, Bamford S, Dawson E, Cole C, et al COSMIC (the Catalogue of Somatic Mutations in Cancer): a resource to investigate acquired mutations in human cancer. Nucleic acids research. 2010;38(Database issue):D652–7. 10.1093/nar/gkp995 19906727 PMC2808858

[3] The Cancer Genome Atlas Network. Comprehensive molecular characterization of human colon and rectal cancer. Nature. 2012;487(7407):330–7. 10.1038/nature11252 22810696 PMC3401966

[4] The Cancer Genome Atlas Research Network. Integrated genomic analyses of ovarian carcinoma. Nature. 2011;474(7353):609–15. 10.1038/nature10166 21720365 PMC3163504

[5] The Cancer Genome Atlas Research Network. Comprehensive genomic characterization of squamous cell lung cancers. Nature. 2012;489(7417):519–25. 10.1038/nature11404 22960745 PMC3466113

[6] The Cancer Genome Atlas Research Network. Genomic and epigenomic landscapes of adult de novo acute myeloid leukemia. The New England journal of medicine. 2013;368(22):2059–74. 10.1056/NEJMoa1301689 . 23634996 PMC3767041

[7] The Cancer Genome Atlas Research Network. Comprehensive molecular characterization of clear cell renal cell carcinoma. Nature. 2013;499(7456):43–9. 10.1038/nature12222 . 23792563 PMC3771322

[8] The Cancer Genome Atlas Research Network, Kandoth C, Schultz N, Cherniack AD, Akbani R, Liu Y, et al Integrated genomic characterization of endometrial carcinoma. Nature. 2013;497(7447):67–73. 10.1038/nature12113 . 23636398 PMC3704730

[9] Flicek P, Amode MR, Barrell D, Beal K, Brent S, Carvalho-Silva D, et al Ensembl 2012. Nucleic acids research. 2012;40(Database issue):D84–90. 10.1093/nar/gkr991 22086963 PMC3245178

[10] Lawrence MS, Stojanov P, Polak P, Kryukov GV, Cibulskis K, Sivachenko A, et al Mutational heterogeneity in cancer and the search for new cancer-associated genes. Nature. 2013 10.1038/nature12213 . 23770567 PMC3919509

[11] De S, Michor F. DNA replication timing and long-range DNA interactions predict mutational landscapes of cancer genomes. Nature biotechnology. 2011;29(12):1103–8. 10.1038/nbt.2030 22101487 PMC3923360

[12] Liu L, De S, Michor F. DNA replication timing and higher-order nuclear organization determine single-nucleotide substitution patterns in cancer genomes. Nature communications. 2013;4:1502 10.1038/ncomms2502 23422670 PMC3633418

[13] Schuster SC, Miller W, Ratan A, Tomsho LP, Giardine B, Kasson LR, et al Complete Khoisan and Bantu genomes from southern Africa. Nature. 2010;463(7283):943–7. 10.1038/nature08795 . 20164927 PMC3890430

[14] Chen X, Chen Z, Chen H, Su Z, Yang J, Lin F, et al Nucleosomes suppress spontaneous mutations base-specifically in eukaryotes. Science. 2012;335(6073):1235–8. 10.1126/science.1217580 . 22403392

[15] Luger K, Mader AW, Richmond RK, Sargent DF, Richmond TJ. Crystal structure of the nucleosome core particle at 2.8 A resolution. Nature. 1997;389(6648):251–60. 10.1038/38444 . 9305837

[16] Segal E, Fondufe-Mittendorf Y, Chen L, Thastrom A, Field Y, Moore IK, et al A genomic code for nucleosome positioning. Nature. 2006;442(7104):772–8. 10.1038/nature04979 16862119 PMC2623244

[17] Schones DE, Cui K, Cuddapah S, Roh TY, Barski A, Wang Z, et al Dynamic regulation of nucleosome positioning in the human genome. Cell. 2008;132(5):887–98. 10.1016/j.cell.2008.02.022 . 18329373 PMC10894452

[18] Mavrich TN, Jiang C, Ioshikhes IP, Li X, Venters BJ, Zanton SJ, et al Nucleosome organization in the Drosophila genome. Nature. 2008;453(7193):358–62. 10.1038/nature06929 18408708 PMC2735122

[19] Kaplan N, Moore IK, Fondufe-Mittendorf Y, Gossett AJ, Tillo D, Field Y, et al The DNA-encoded nucleosome organization of a eukaryotic genome. Nature. 2009;458(7236):362–6. 10.1038/nature07667 19092803 PMC2658732

[20] Valouev A, Johnson SM, Boyd SD, Smith CL, Fire AZ, Sidow A. Determinants of nucleosome organization in primary human cells. Nature. 2011;474(7352):516–20. 10.1038/nature10002 21602827 PMC3212987

[21] Yen K, Vinayachandran V, Batta K, Koerber RT, Pugh BF. Genome-wide nucleosome specificity and directionality of chromatin remodelers. Cell. 2012;149(7):1461–73. 10.1016/j.cell.2012.04.036 22726434 PMC3397793

[22] Ernst J, Kheradpour P, Mikkelsen TS, Shoresh N, Ward LD, Epstein CB, et al Mapping and analysis of chromatin state dynamics in nine human cell types. Nature. 2011;473(7345):43–9. 10.1038/nature09906 21441907 PMC3088773

[23] Tolstorukov MY, Volfovsky N, Stephens RM, Park PJ. Impact of chromatin structure on sequence variability in the human genome. Nature structural & molecular biology. 2011;18(4):510–5. 10.1038/nsmb.2012 21399641 PMC3188321

[24] Warnecke T, Becker EA, Facciotti MT, Nislow C, Lehner B. Conserved substitution patterns around nucleosome footprints in eukaryotes and Archaea derive from frequent nucleosome repositioning through evolution. PLoS computational biology. 2013;9(11):e1003373 10.1371/journal.pcbi.1003373 24278010 PMC3836710

[25] Chen K, Xi Y, Pan X, Li Z, Kaestner K, Tyler J, et al DANPOS: dynamic analysis of nucleosome position and occupancy by sequencing. Genome research. 2013;23(2):341–51. 10.1101/gr.142067.112 23193179 PMC3561875

[26] Consortium EP, Dunham I, Kundaje A, Aldred SF, Collins PJ, Davis CA, et al An integrated encyclopedia of DNA elements in the human genome. Nature. 2012;489(7414):57–74. 10.1038/nature11247 22955616 PMC3439153

[27] Lister R, Pelizzola M, Dowen RH, Hawkins RD, Hon G, Tonti-Filippini J, et al Human DNA methylomes at base resolution show widespread epigenomic differences. Nature. 2009;462(7271):315–22. 10.1038/nature08514 19829295 PMC2857523

[28] Yu M, Hon GC, Szulwach KE, Song CX, Zhang L, Kim A, et al Base-resolution analysis of 5-hydroxymethylcytosine in the mammalian genome. Cell. 2012;149(6):1368–80. 10.1016/j.cell.2012.04.027 22608086 PMC3589129

[29] The 1000 Genomes Project Consortium, Abecasis GR, Auton A, Brooks LD, DePristo MA, Durbin RM, et al An integrated map of genetic variation from 1,092 human genomes. Nature. 2012;491(7422):56–65. 10.1038/nature11632 23128226 PMC3498066

[30] Kong A, Frigge ML, Masson G, Besenbacher S, Sulem P, Magnusson G, et al Rate of de novo mutations and the importance of father's age to disease risk. Nature. 2012;488(7412):471–5. 10.1038/nature11396 22914163 PMC3548427

[31] Sherry ST, Ward MH, Kholodov M, Baker J, Phan L, Smigielski EM, et al dbSNP: the NCBI database of genetic variation. Nucleic acids research. 2001;29(1):308–11. 11125122 10.1093/nar/29.1.308 PMC29783

[32] Gaffney DJ, McVicker G, Pai AA, Fondufe-Mittendorf YN, Lewellen N, Michelini K, et al Controls of nucleosome positioning in the human genome. PLoS genetics. 2012;8(11):e1003036 10.1371/journal.pgen.1003036 23166509 PMC3499251

[33] Weghorn D, Lassig M. Fitness landscape for nucleosome positioning. Proceedings of the National Academy of Sciences of the United States of America. 2013;110(27):10988–93. 10.1073/pnas.1210887110 . 23784778 PMC3704022

[34] North JA, Shimko JC, Javaid S, Mooney AM, Shoffner MA, Rose SD, et al Regulation of the nucleosome unwrapping rate controls DNA accessibility. Nucleic acids research. 2012;40(20):10215–27. 10.1093/nar/gks747 22965129 PMC3488218

[35] Shim EY, Hong SJ, Oum JH, Yanez Y, Zhang Y, Lee SE. RSC mobilizes nucleosomes to improve accessibility of repair machinery to the damaged chromatin. Molecular and cellular biology. 2007;27(5):1602–13. 10.1128/MCB.01956-06 17178837 PMC1820475

[36] Brogaard K, Xi L, Wang JP, Widom J. A map of nucleosome positions in yeast at base-pair resolution. Nature. 2012;486(7404):496–501. 10.1038/nature11142 . 22722846 PMC3786739

[37] Lang GI, Parsons L, Gammie AE. Mutation rates, spectra, and genome-wide distribution of spontaneous mutations in mismatch repair deficient yeast. G3. 2013;3(9):1453–65. 10.1534/g3.113.006429 23821616 PMC3755907

[38] Gammie AE, Erdeniz N, Beaver J, Devlin B, Nanji A, Rose MD. Functional characterization of pathogenic human MSH2 missense mutations in Saccharomyces cerevisiae. Genetics. 2007;177(2):707–21. 10.1534/genetics.107.071084 17720936 PMC2034637

[39] Leroy JL, Kochoyan M, Huynh-Dinh T, Gueron M. Characterization of base-pair opening in deoxynucleotide duplexes using catalyzed exchange of the imino proton. Journal of molecular biology. 1988;200(2):223–38. . 2836594 10.1016/0022-2836(88)90236-7

[40] Duguid JG, Bloomfield VA, Benevides JM, Thomas GJ Jr. DNA melting investigated by differential scanning calorimetry and Raman spectroscopy. Biophys J. 1996;71(6):3350–60. 10.1016/S0006-3495(96)79528-0 8968604 PMC1233822

[41] Chodavarapu RK, Feng S, Bernatavichute YV, Chen PY, Stroud H, Yu Y, et al Relationship between nucleosome positioning and DNA methylation. Nature. 2010;466(7304):388–92. 10.1038/nature09147 20512117 PMC2964354

[42] Johnson SM, Tan FJ, McCullough HL, Riordan DP, Fire AZ. Flexibility and constraint in the nucleosome core landscape of Caenorhabditis elegans chromatin. Genome research. 2006;16(12):1505–16. 10.1101/gr.5560806 17038564 PMC1665634

[43] Shen JC, Rideout WM 3rd, Jones PA. The rate of hydrolytic deamination of 5-methylcytosine in double-stranded DNA. Nucleic acids research. 1994;22(6):972–6. 8152929 10.1093/nar/22.6.972 PMC307917

[44] Lynch M. Rate, molecular spectrum, and consequences of human mutation. Proceedings of the National Academy of Sciences of the United States of America. 2010;107(3):961–8. 10.1073/pnas.0912629107 20080596 PMC2824313

[45] Tang Y, Gao XD, Wang Y, Yuan BF, Feng YQ. Widespread existence of cytosine methylation in yeast DNA measured by gas chromatography/mass spectrometry. Anal Chem. 2012;84(16):7249–55. 10.1021/ac301727c . 22852529 PMC4706227

[46] Cadet J, Douki T, Ravanat JL. Oxidatively generated damage to the guanine moiety of DNA: mechanistic aspects and formation in cells. Acc Chem Res. 2008;41(8):1075–83. 10.1021/ar700245e . 18666785

[47] Cheng KC, Cahill DS, Kasai H, Nishimura S, Loeb LA. 8-Hydroxyguanine, an abundant form of oxidative DNA damage, causes G——T and A——C substitutions. The Journal of biological chemistry. 1992;267(1):166–72. . 1730583

[48] Kamiya H, Kasai H. Formation of 2-hydroxydeoxyadenosine triphosphate, an oxidatively damaged nucleotide, and its incorporation by DNA polymerases. Steady-state kinetics of the incorporation. The Journal of biological chemistry. 1995;270(33):19446–50. . 7642627 10.1074/jbc.270.33.19446

[49] Yang XL, Sugiyama H, Ikeda S, Saito I, Wang AH. Structural studies of a stable parallel-stranded DNA duplex incorporating isoguanine:cytosine and isocytosine:guanine basepairs by nuclear magnetic resonance spectroscopy. Biophys J. 1998;75(3):1163–71. 10.1016/S0006-3495(98)74035-4 9726918 PMC1299791

[50] Langley SA, Karpen GH, Langley CH. Nucleosomes shape DNA polymorphism and divergence. PLoS genetics. 2014;10(7):e1004457 10.1371/journal.pgen.1004457 24991813 PMC4081404

[51] Kenigsberg E, Bar A, Segal E, Tanay A. Widespread compensatory evolution conserves DNA-encoded nucleosome organization in yeast. PLoS computational biology. 2010;6(12):e1001039 10.1371/journal.pcbi.1001039 21203484 PMC3009600

[52] West JA, Cook A, Alver BH, Stadtfeld M, Deaton AM, Hochedlinger K, et al Nucleosomal occupancy changes locally over key regulatory regions during cell differentiation and reprogramming. Nature communications. 2014;5:4719 10.1038/ncomms5719 25158628 PMC4217530

[53] Polak P, Karlic R, Koren A, Thurman R, Sandstrom R, Lawrence MS, et al Cell-of-origin chromatin organization shapes the mutational landscape of cancer. Nature. 2015;518(7539):360–4. 10.1038/nature14221 . 25693567 PMC4405175

[54] Nahkuri S, Taft RJ, Mattick JS. Nucleosomes are preferentially positioned at exons in somatic and sperm cells. Cell cycle. 2009;8(20):3420–4. . 19823040 10.4161/cc.8.20.9916

[55] Andersson R, Enroth S, Rada-Iglesias A, Wadelius C, Komorowski J. Nucleosomes are well positioned in exons and carry characteristic histone modifications. Genome research. 2009;19(10):1732–41. 10.1101/gr.092353.109 19687145 PMC2765275

[56] Thomson JA, Itskovitz-Eldor J, Shapiro SS, Waknitz MA, Swiergiel JJ, Marshall VS, et al Embryonic stem cell lines derived from human blastocysts. Science. 1998;282(5391):1145–7. . 9804556 10.1126/science.282.5391.1145

[57] Aird D, Ross MG, Chen WS, Danielsson M, Fennell T, Russ C, et al Analyzing and minimizing PCR amplification bias in Illumina sequencing libraries. Genome biology. 2011;12(2):R18 10.1186/gb-2011-12-2-r18 21338519 PMC3188800

[58] Cokus SJ, Feng S, Zhang X, Chen Z, Merriman B, Haudenschild CD, et al Shotgun bisulphite sequencing of the Arabidopsis genome reveals DNA methylation patterning. Nature. 2008;452(7184):215–9. 10.1038/nature06745 18278030 PMC2377394

[59] Langmead B, Salzberg SL. Fast gapped-read alignment with Bowtie 2. Nature methods. 2012;9(4):357–9. 10.1038/nmeth.1923 22388286 PMC3322381

[60] Li H, Handsaker B, Wysoker A, Fennell T, Ruan J, Homer N, et al The Sequence Alignment/Map format and SAMtools. Bioinformatics. 2009;25(16):2078–9. 10.1093/bioinformatics/btp352 19505943 PMC2723002

[61] Allan J, Fraser RM, Owen-Hughes T, Keszenman-Pereyra D. Micrococcal nuclease does not substantially bias nucleosome mapping. Journal of molecular biology. 2012;417(3):152–64. 10.1016/j.jmb.2012.01.043 22310051 PMC3314939

[62] Quinlan AR, Hall IM. BEDTools: a flexible suite of utilities for comparing genomic features. Bioinformatics. 2010;26(6):841–2. 10.1093/bioinformatics/btq033 20110278 PMC2832824

[63] Meyer LR, Zweig AS, Hinrichs AS, Karolchik D, Kuhn RM, Wong M, et al The UCSC Genome Browser database: extensions and updates 2013. Nucleic acids research. 2013;41(Database issue):D64–9. 10.1093/nar/gks1048 23155063 PMC3531082

[64] Danecek P, Auton A, Abecasis G, Albers CA, Banks E, DePristo MA, et al The variant call format and VCFtools. Bioinformatics. 2011;27(15):2156–8. 10.1093/bioinformatics/btr330 21653522 PMC3137218

[65] Pruitt KD, Tatusova T, Brown GR, Maglott DR. NCBI Reference Sequences (RefSeq): current status, new features and genome annotation policy. Nucleic acids research. 2012;40(Database issue):D130–5. 10.1093/nar/gkr1079 22121212 PMC3245008

[66] McKenna A, Hanna M, Banks E, Sivachenko A, Cibulskis K, Kernytsky A, et al The Genome Analysis Toolkit: a MapReduce framework for analyzing next-generation DNA sequencing data. Genome research. 2010;20(9):1297–303. 10.1101/gr.107524.110 20644199 PMC2928508

[67] DePristo MA, Banks E, Poplin R, Garimella KV, Maguire JR, Hartl C, et al A framework for variation discovery and genotyping using next-generation DNA sequencing data. Nature genetics. 2011;43(5):491–8. 10.1038/ng.806 21478889 PMC3083463

[68] Van der Auwera GA, Carneiro MO, Hartl C, Poplin R, Del Angel G, Levy-Moonshine A, et al From FastQ data to high confidence variant calls: the Genome Analysis Toolkit best practices pipeline. Curr Protoc Bioinformatics. 2013;11(1110):11 0 1–0 33. 10.1002/0471250953.bi1110s43 25431634 PMC4243306

[69] Li H, Durbin R. Fast and accurate short read alignment with Burrows-Wheeler transform. Bioinformatics. 2009;25(14):1754–60. 10.1093/bioinformatics/btp324 19451168 PMC2705234

[70] Leinonen R, Sugawara H, Shumway M, International Nucleotide Sequence Database Collaboration. The sequence read archive. Nucleic acids research. 2011;39(Database issue):D19–21. 10.1093/nar/gkq1019 21062823 PMC3013647

[71] Koboldt DC, Zhang Q, Larson DE, Shen D, McLellan MD, Lin L, et al VarScan 2: somatic mutation and copy number alteration discovery in cancer by exome sequencing. Genome research. 2012;22(3):568–76. 10.1101/gr.129684.111 22300766 PMC3290792

