# Supplementary figures and images for: Increasing Nucleosome Occupancy Is Correlated with an Increasing Mutation Rate so Long as DNA Repair Machinery Is Intact
Source: PLoS One. 2015 Aug 26;10(8):e0136574. doi: 10.1371/journal.pone.0136574 (PMC4550472; doi:10.1371/journal.pone.0136574)

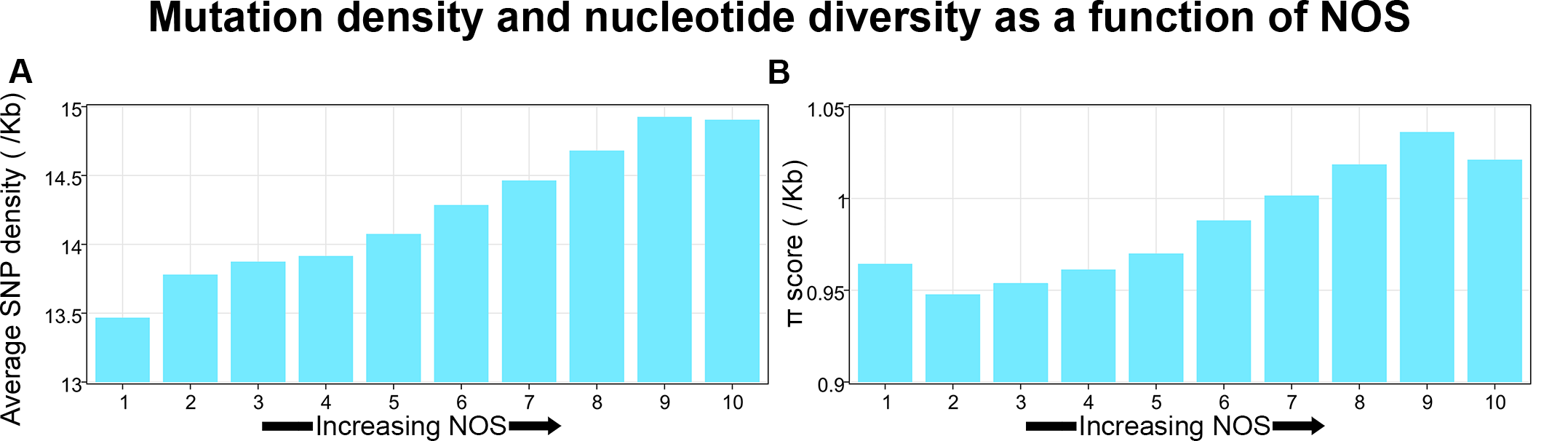

Supplement: S1 Fig — A, The average SNP density was calculated as the number of SNPs per 1,000 bp then averaged for each equal sized group corresponding to increasing nucleosome occupancy. Genetic variation data was generated from The 1000 Genomes Project. B, Groups 1–10 correspond to groups with increasing nucleosome occupancy scores (NOS). The π score is a measure of nucleotide diversity and was calculated in 1,000 bp bins. (TIF) [file pone.0136574.s001.tif]

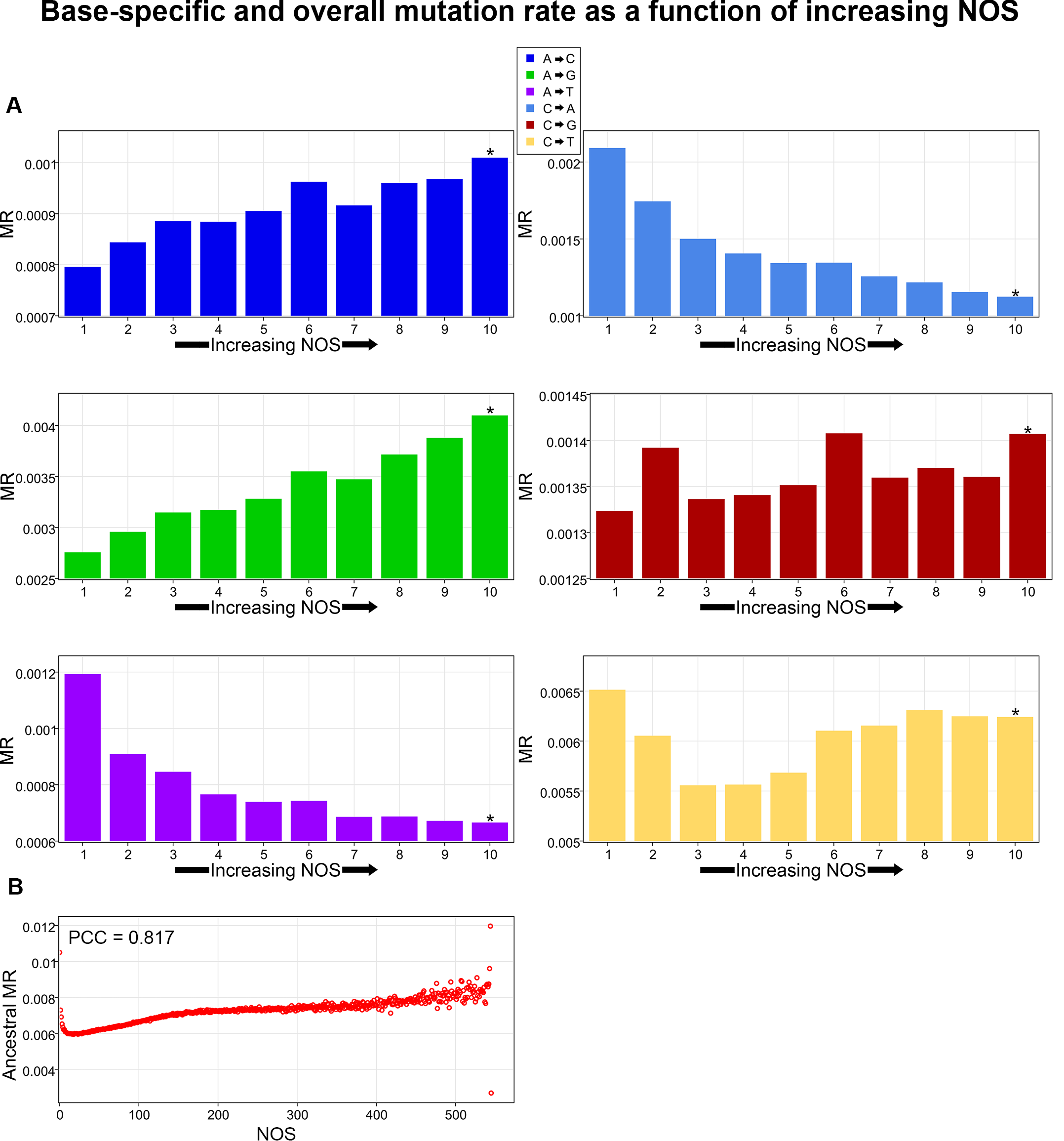

Supplement: S2 Fig — A, Ancestral base-specific mutation rates (MR) calculated for ten equally sized groups corresponding to increasing nucleosome occupancy scores (NOS) with color coded legend for the type of mutation at top, with asterisks denoting statistical significance (p-value < 0.01) between the first and last group. B, Ancestral MR in relation to nucleosome occupancy with a Pearson’s correlation coefficient (PCC) of 0.817. (TIF) [file pone.0136574.s002.tif]

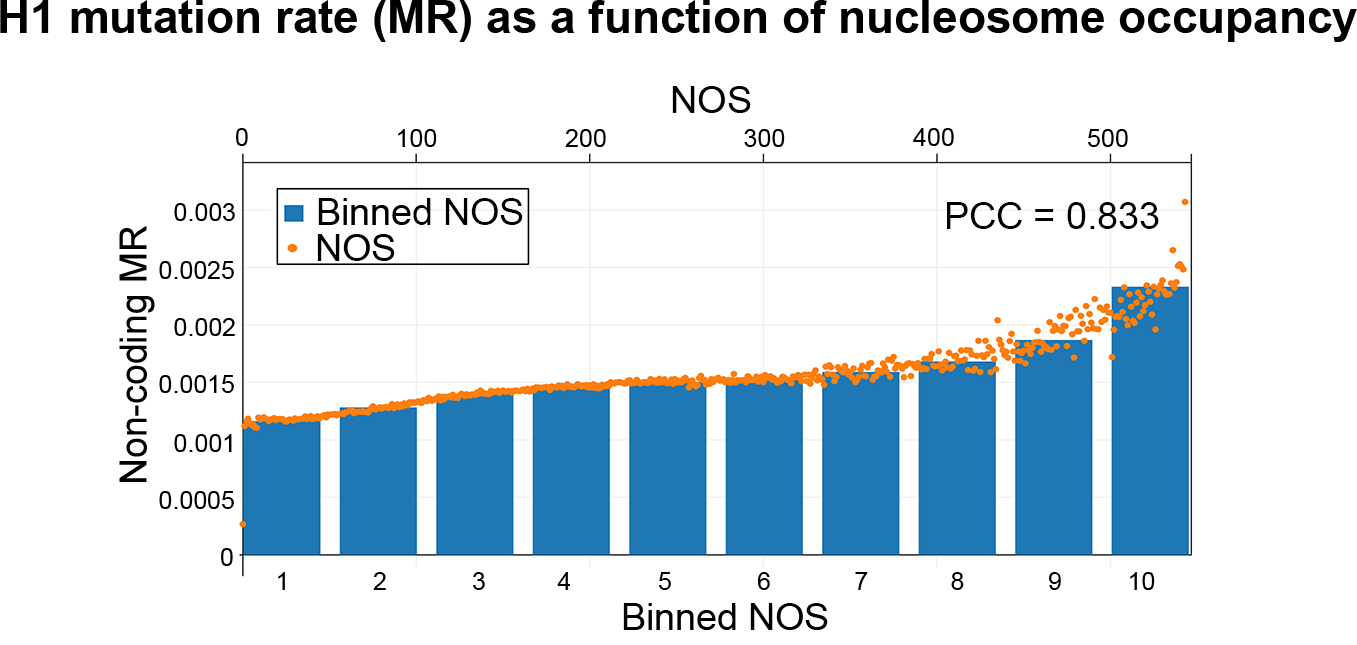

Supplement: S3 Fig — Bottom x-axis corresponds to the bar graph depicting the NOS for 10 equally sized groups of increasing nucleosome occupancy. Top x-axis corresponds to the scatter plot depiction of the same data for each individual NOS. Pearson’s correlation coefficient (PCC) of 0.833. (TIF) [file pone.0136574.s003.tif]
